# Community place attachment as a significant correlate of motivation to change substance use behavior among adults who inject drugs in rural U.S.

**DOI:** 10.3389/fpubh.2026.1746203

**Published:** 2026-03-25

**Authors:** Janet K. Otachi, Shawndaya S. Thrasher, Keith J. Watts, Juma S. Mwenda, Hilary L. Surratt

**Affiliations:** 1 School of Social Work and Family Science, University of Louisville, Louisville, KY, United States; 2 School of Social Work, Louisiana State University, Baton Rouge, LA, United States; 3 College of Social Work, University of Kentucky, Lexington, KY, United States; 4 Department of Behavioral Science, University of Kentucky, Lexington, KY, United States

**Keywords:** belongingness, community place attachment, connectedness, PWID, substance use behavior change

## Abstract

**Introduction:**

While motivation is critical for substance use disorder (SUD) recovery, many individuals fail to engage in treatment. The need to belong is a fundamental human motivation, yet the role of community place attachment (CPA) as a social asset in fostering motivation to change substance use behaviors (MCSUB) among people who inject drugs (PWID) is understudied. This paper examines the effect of CPA on PWIDs’ MCSUB.

**Methodology:**

Cross-sectional data were collected from 350 PWID recruited from syringe service programs in rural Appalachian Kentucky (2018–2021). We used hierarchical multivariable linear regression to assess the association between CPA and MCSUB, controlling for socio-ecologic covariates including socio-demographics, mental health disorders (MHD), substance use stigma (SUS), violence experiences (VE), and substance use treatment utilization (SUTU).

**Key findings:**

A majority of participants (68.0%) reported high MCSUB, yet only 34.4% reported past-year SUTU, revealing a significant “motivation-treatment gap.” In the final model, stronger CPA was significantly associated with higher MCSUB (*β* = 0.155, *p* = 0.009).

**Conclusion:**

Fulfilling the need to belong through community place attachment may be a key social asset that enhances internal motivation for recovery among PWID. However, this motivation is often insufficient to overcome barriers presented by co-occurring MHD and trauma. Findings underscore the importance of asset-based approaches that strengthen community place attachment/ identity or belongingness. Integrating care for substance use disorders, mental health disorders, and trauma-informed care in tailored interventions and policies for PWID may further enhance motivation to change substance use behavior and support translation of motivation into action.

## Introduction

People who inject drugs (PWID) experience disparities in substance use disorders (SUDs) and related adverse physical and mental health outcomes (1, 2). Evidence suggests that individuals with SUDs, particularly PWID, experience greater social isolation and fewer social connections than those without SUD. Substance use stigma (SUS) contributes to this isolation and increases susceptibility to risky substance use behaviors (e.g., fentanyl use and its analogs) and adverse outcomes (e.g., drug-induced overdose (OD) deaths) (1, 3, 4).

According to the social capital perspective, community place attachment or identity (i.e., subjective experiences or feelings of deep connection with social groups and physical places) (5, 6) is a modifiable factor that plays a critical role in SUD treatment engagement and recovery (6–8), by increasing an individual’s motivation to abstain from substance use (9, 10). Evidence suggests that attachment to one’s hometown, even after moving to other places later in adulthood, can be an indicator of CPA, with feelings of connection to one’s hometown later in life associated with an increased likelihood of maintaining communication and strong ties with their hometown, resulting in greater social support and a strong sense of belongingness (5, 11). Therefore, fostering CPA among PWID may enhance their treatment engagement and SUD recovery (5, 12–19). Recovery involves restoring a meaningful sense of CPA or belongingness and fostering a positive sense of identity separate from one’s condition, while rebuilding one’s life despite, or within, the limitations of their condition (20). Thus, enhancing CPA can significantly support PWID in their recovery journey (21).

Evidence further suggests that a sense of CPA increases personal capital, including resilience, coping skills, self-efficacy. These factors may enhance motivation to engage in substance use treatment through social interactions that promote recovery and well-being (9, 19, 22). For individuals in recovery, developing personal capital depends not only on their individual efforts but also on fostering a sense of belonging or attachment to a place (CPA) (22). Additionally, positive social interactions with providers have also been shown to enhance SUD treatment engagement, adherence, and favorable outcomes (e.g., long-term sobriety and improved mental and physical health) (23–25). Thus, highlighting the importance of addressing substance use stigma or SUS, which impedes SUD recovery (4, 26–28).

Despite evidence suggesting the importance of CPA in enhancing motivation to change substance use behavior and treatment engagement, fewer studies have examined its role in promoting substance use recovery (29), particularly among PWID. Most research has focused on CPA in the context of social support, indicating that a greater sense of social support is associated with substance use treatment engagement and a reduced likelihood of relapse (6). However, the specific influence of CPA on motivation to engage in substance use treatment remains underexplored. The primary aim of this paper is to examine the role of CPA on PWIDs’ motivation to change substance use behavior. We hypothesized that increased CPA would increase PWIDs’ perceived importance in cutting down or stopping substance use. Various factors, including mental health disorders, experiences of all forms of violence and related trauma, and gaps in treatment utilization or engagement, have been shown to reduce motivation to change substance use behavior and engage in SUD treatment, and PWID are disproportionately affected by these factors (30–34). Some studies show that factors such as gender (35) and age (31) significantly influence motivation to engage in substance use treatment. Additionally, high rates of treatment non-adherence and dropout have been reported among young or emerging adults compared to their older counterparts (36–38). Given the existing literature supporting these factors as significant socio-ecologic correlates of motivation to change substance use behaviors and SUD treatment utilization, we controlled for these factors.

## Materials and methods

### Research design

This cross-sectional study included 35–40-min face-to-face interviews with a voluntary sample (*n* = 350) of PWID. Data were collected from a mixed-methods study aimed at identifying the socio-ecological factors influencing utilization of newly implemented syringe service programs (SSPs) among people who inject drugs (PWID) in rural Appalachian Kentucky.

### Study area

Participants were recruited from geographically dispersed syringe service programs (SSPs) within local health departments and community-based locations serving PWID in Kentucky’s Appalachian region from 2018 to 2021. Eligible participants were required to be at least 18 years old and current substance users, having injected drugs, either one type or multiple types within the past 30 days. The brief GAIN Screener (39) was used to collect information on demographics, substance use behaviors, mental health disorders, and the environment.

### Sampling and data collection

The response driven sample technique (RDS) was utilized for study recruitment (40) and supplemented with direct community outreach. Participants received a $20 gift card as reimbursement for their study participation and transportation after completing the face-to-face interviews. Ethical concerns about fairness, autonomy, and potential coercion were considered when determining the incentive. The University’s Institutional Review Board approved the study.

### Study measures

#### Motivation to change substance use behavior (MCSUB)

Motivation related to changing substance use behavior is a complex phenomenon that includes both intrinsic and extrinsic factors. Intrinsic motivation refers to internal feelings such as the personal desire to cut down or stop substance use, while extrinsic motivation involves external influences that affect one’s confidence in their ability to cut down or stop substance use amidst external challenges (41). In assessing MCSUB, we focused on intrinsic motivation which has been shown to produce long-term outcomes than extrinsic motivation (42), whereby participants were asked to rate how important it was for them to cut down or stop substance use on a scale from 0 to 10, with 0 being “not important” and 10 being “extremely important.” Higher scores indicated greater MCSUB (43). For descriptive analysis, this measure was also categorized as Low (0–4), Medium (5–7), and High (8–10) MCSUB (see Table 1).

**Table 1:** Participant characteristics (*N* = 350).

PWID characteristics	N (%) or M (SD)
Demographics	
Age in years [mean (SD)]	37.6 (9.6)
Gender (female)	169 (48.3%)
County	
Clark	164 (46.9%)
Knox	143 (40.9%)
Owsley	43 (12.3%)
Mental health	
Past year severe mental health distress (MHD)	293 (84.0%)
Lifetime substance use stigma (SUS) from Family and/ or Provider	315 (90.0%)
Experiences of violence	
Past year violence experiences (VE) of any form	179 (51.1%)
Belongingness or connectedness	
Rural hometown	280 (80.2%)
Mean community place attachment (CPA) [mean (SD)]	3.25 (1.3)
Low CPA (0–3)	145 (52.0%)
High CPA (>3)	134 (48.0%)
SUD treatment motivation and engagement	
Motivation to change substance use behavior (MCSUB): Importance to cut down/stop substance use [mean (SD)]	7.9 (3.0)
Low (0–4)	43 (12.3%)
Medium (5–7)	69 (19.7%)
High (8–10)	238 (68.0%)
Substance Use Treatment Utilization (SUTU) in the past year	120 (34.4%)

#### Community place attachment (CPA)

Participants were asked to name the place where they were raised for the majority of their life (i.e., their hometown), and whether they considered the place to be a rural community (0 = no; 1 = yes). Assessment of belongingness or connectedness was conducted among PWID who considered the place where they were raised for the majority of their life (i.e., their hometown) as rural (*n* = 280) utilizing the CPA scale, adapted from a 6-item place attachment scale (*α* = 0.929) (44). Participants were asked to rate on a scale of 1 “strongly agree” to 5 “strongly disagree” whether their hometown means a lot to them, whether they are very attached to their home town, whether they have a lot of fond memories of their home town, whether their hometown is special to them, whether they strongly identify with their hometown, and whether they feel that their hometown is part of them. Mean CPA scores (*M* = 3.25; SD = 1.3) were obtained for analysis (see Table 1), and this measure was also categorized as low CPA (0–3) and high CPA (>3).

#### Mental health disorders (MHD)

Mental health disorders was assessed using 6 scale items (*α* = 0.717) obtained from the GAIN brief screener that assessed somatic complaints, depressive symptoms, anxious thoughts, traumatic experiences, psychosis, and suicidality. Study participants self-reported their experiences of MHD more than a year ago, 4–12 months ago, 2–3 months ago, during the past 30 days, or never. For analysis, we included the past year MHD with scores of 3 and above (3+), indicating severe MHD requiring mental health services. This measure was categorized as “severe MHD in the past year (0=no; 1=yes)”.

#### Substance use disorders (SUD)

Substance use disorders was assessed using an 11-items scale (α = 0.871) from the GAIN short screener and included symptoms of substance abuse, dependence, and craving as per the Diagnostic and Statistical Manual of Mental Disorders, 5th edition (39). Participants self-reported their experiences of SUD in the past 30 days, past 3 months, past year, and lifetime. For analysis, we included past-year SUD with scores of 3 and above (3+), indicating severe SUD requiring interventions/treatment services. This measure was categorized as “severe substance use disorder in the past year (0 = no; 1 = yes)”.

#### Substance use stigma (SUS)

The 6-item (α = 0.828) Substance Use Stigma Mechanisms Scale (45) assessed self-reported enacted substance use stigma (SUS) by family and/ or providers. The scale included 3 items to measure family stigma and 3 items to measure provider stigma, each on a 5-point Likert scale ranging from “Never” to “Often.” Our analysis dichotomized this measure to assess the lifetime history of stigma from family and/or providers (0 = no; 1 = yes).

#### Substance use treatment utilization (SUTU)

Substance use treatment utilization was assessed by asking participants to self-report the last time they received any counseling, treatment, medication, case management, aftercare for substance use. Responses included never, more than 12 months ago, 4 to 12 months ago, 1 to 3 months ago, and within the past week. For analysis, we included SUTU within the past year (0 = no; 1 = yes).

#### Violence experiences (VE)

We utilized four items from the GAIN General Victimization Scale (α = 0.682) to assess PWID’s past year experience of violence (being attacked with a weapon, physical abuse, sexual abuse, and emotional abuse). Our analysis dichotomized this measure to assess past-year experiences of any violence (0 = no; 1 = yes).

#### Demographics

Age was self-reported in years, and gender was categorized as female vs. male.

## Data analysis

Data analysis included responses from a sample of 350 PWID. Missing data for all variables were examined and excluded from the analysis (i.e., one case for MHD, SUTU, and rural hometown, and three cases for the CPA scale). First, for descriptive analysis, we derived descriptive statistics for the study measures, including means with standard deviations for continuous variables and proportions for categorical variables. Second, a hierarchical multivariable linear regression (HMLR) analysis was conducted to assess the association between CPA and MCSUB, controlling for age, gender, past-year severe MHD, SUTU, and VE. A hierarchical multivariable linear regression analysis is a statistical method that is utilized to assess the associations between specific independent and dependent variables, and can help determine if certain theoretical concepts or perspectives demonstrate the associations that they suggest (46). In our regression analysis, we assessed the social capital perspective’s assertion that CPA is a key determinant of an individual’s MCSUB, which subsequently leads to engagement in substance use treatment. In step 1 of our analysis, we included key socio-ecologic predictors of MCSUB, including age, gender, past-year severe MHD, SUTU, and VE. In step 2, we added CPA as a predictor of MCSUB. Our goal was to determine if the regression model would change when controlling for socio-ecologic predictors of MCSUB in step 1 and adding CPA in step 2. Results highlighting step 1 and step 2 outputs are summarized in Table 2. All analyses were conducted using SPSS IBM Statistics version 30, with a *p*-value of less than 0.05 indicating statistical significance.

**Table 2:** Hierarchical multivariable linear regression analysis predicting PWIDs importance to cut down or stop substance use.

**Variable**	**Step 1**				**Step 2**			
	Β	**Std. error** **SE B**	***β***	***p*-value**	Β	**Std. error** **SE B**	***β***	***p-*value**
Age	0.007	0.018	0.022	0.709	0.004	0.018	0.012	0.841
Gender (female); Male, referent	0.330	0.353	0.056	0.350	0.423	0.351	0.072	0.228
Past year severe MHD (Yes); No, referent	1.143	0.487	**0.142**	0.020*	1.193	0.482	**0.148**	0.014*
Lifetime SUS (Yes); No, referent	−0.393	0.601	−0.040	0.514	−0.298	0.596	−0.030	0.618
Past year VE (Yes); No, referent	−0.730	0.350	**−0.123**	0.038*	−0.723	0.346	**−0.122**	0.038*
SUTU in the past year (Yes); No, referent	0.907	0.372	**0.146**	**0.016**	0.854	0.369	**0.137**	0.021*
Mean (CPA)	–	–	–	–	0.349	0.133	**0.155**	0.009*
*R* ^2^		0.060				0.083		
Adjusted *R*^2^		0.039				0.059		
*R*^2^ change		0.060				0.023		
*F* change		2.859				6.884		
*P-*value		0.010*				0.009*		

*p-value of *p* < 0.05 indicates statistical significance. Bolded values signify variables that are statistically significantly associated with polyvictimization.

## Results

### Participant characteristics

As highlighted in Table 1, approximately 48% of our sample of PWID were females, with an average age of about 38 years. In the past year, 84.0% of participants reported MHD, and 90% reported lifetime SUS from family and/or providers. Stigma was more commonly reported from family (86.6%) than from providers (66.9%). However, both experiences of enacted stigma from family and providers were still significantly high. Our analysis of the MCSUB highlighted that a majority of our PWID sample felt that it was important to cut down or stop using substances (*M* = 7.9; SD = 3.0). Despite high scores in MCSUB (68.0%), only about a third of our sample (34.4%) reported SUTU in the past year (see Table 1). Our findings also indicated that slightly more than half (52.0%) reported a low sense of CPA toward their hometown (i.e., the place where they grew up or spent most of their lives).

### Factors associated with PWIDs’ motivation to change substance use behavior

Findings from our HMLR analysis (see Table 2), which included age, gender, severe MHD, VE, and SUTU in the past year, as well as lifetime SUS, in step 1, explained 6.0% of the variance in the motivation to change substance use behaviors (MCSUB). Past year MHD (*β* = 0.142, *p* = 0.020), VE (*β* = −0.123, *p* = 0.038), and SUTU (*β* = 0.146, *p* = 0.020) were significantly associated with motivation to change substance use behavior (MCSUB) in step 1. Community place attachment or CPA was entered in step 2, explaining 8.3% of the variance in the MCSUB [*F* (1, 270) = 6.884, *p* = 0.009]. Past year MHD (*β* = 0.148, *p* = 0.014), VE (*β* = −0.122, *p* = 0.038), and SUTU (*β* = 0.137, *p* = 0.021) remained significantly associated with PWID’s MCSUB in step 2. Additionally, CPA (*β* = 0.155, *p* = 0.009) significantly predicted motivation to change substance use behavior. Based on our findings, CPA was the strongest predictor of PWIDs’ MCSUB. Lastly, lifetime substance use stigma (SUS), gender, and age were not significant predictors of MCSUB (*p* > 0.05) in steps 1 and 2 of the HMLR analysis.

## Discussion

The primary objective of this paper was to examine the association between community place attachment (CPA) and motivation to change substance use behavior (MCSUB) among people who inject drugs (PWID) in rural Appalachian Kentucky. Our findings support the central hypothesis that stronger CPA is significantly associated with a higher perceived importance of cutting down or stopping substance use among PWID. This association remained significant even after controlling for demographics, mental health, experiences of violence, and substance use factors, which are known to correlate with MCSUB. This underscores the unique role of CPA as an asset in supporting PWIDs’ intrinsic motivation to change substance use behavior. Thus, our results suggest that enhancing community identity or place attachment can significantly contribute to substance use recovery capital. Encouraging the utilization of community harm reduction settings (47–51), which provides a stigma-free environment for PWID, may enhance feelings of belongingness and place identity in this vulnerable population, supporting their substance use recovery and engagement in the care continuum (52–55).

A key finding of this study is the significant disconnect between participants’ perceived sense of motivation to change substance use behavior and their uptake of substance use treatment services in the past year. While over two-thirds of the participants reported high importance in cutting down or stopping substance use, only about one-third reported engagement in substance use treatment within the past year. This ‘motivation-treatment gap’ suggests that while intrinsic motivation is essential, extrinsic socio-structural barriers prevalent in rural Appalachian regions may hinder SUTU among vulnerable PWID (2, 56, 57). These barriers include long distance to community substance use treatment facilities, lack of transportation and health insurance coverage, and challenges in navigating complex healthcare systems (2, 56, 57). Though we found a significant association between greater experiences of severe MHD in the past year and higher motivation to change their substance use behavior, untreated MHD among PWID may impede the ability to initiate and maintain engagement with substance use treatment services (58–60). Thus, the lower prevalence of SUD treatment engagement in the past year observed in our sample of PWID may reflect the impact of untreated mental health conditions. Nevertheless, the complexity of the interaction between co-occurring substance use and mental illness can significantly influence an individual’s motivation to change substance use behavior. For instance, during a mental health crisis, an individual may feel compelled to comply with substance use treatment during hospitalization to address the acute psychiatric symptoms but often discontinue treatment post-discharge (61, 62). Conversely, severe MHD and SUD can impair decision-making skills regarding the need for treatment (63). Thus, the interaction between untreated comorbid mental health conditions and substance use disorders, as well as how these factors affect substance use treatment utilization warrants further research.

Experiences of violence of all forms in the past year emerged as a significant predictor of motivation to change substance use behavior. PWID with experiences of violence in the past year reported lower motivation to change substance use behavior. This is consistent with literature demonstrating that trauma and adverse mental health outcomes from multiple experiences of violence or polyvictimization among PWID can foster feelings of hopelessness and serve as a direct trigger for continued substance use as a negative coping mechanism (32, 64–68). The present study adds to this by highlighting that trauma from experiences of violence may also directly diminish the foundational belief that change is important, necessitating the integration of trauma-informed care principles into all prevention and treatment efforts for this vulnerable population, to enhance their motivation to change substance use behavior.

Notably, our findings indicated that PWID with a lifetime history of experiencing substance use stigma (SUS) were more likely to utilize treatment services. One possible explanation is that family stigma, which was more prevalent in our sample (86.6% vs. 66.9% from providers), may create external pressure or a desire to mend relationships that motivates treatment seeking. However, this interpretation requires further empirical investigation. Our findings further indicate that PWIDs not only face stigma from family but are also at risk of experiencing stigma from healthcare providers. Additionally, they may encounter other intersecting stigmas (e.g., internalized stigma, condition stigma, including stigma from injecting drug use and other comorbidities such as mental illnesses, and anticipated stigma from others due to community prejudices related to injecting drug use) which may significantly contribute to the healthcare disparities that they face (4, 59, 69–74). This highlights the urgent need to create stigma-free care environments and advocate for stigma-free communities to address the disparities in substance use and adverse outcomes faced by this vulnerable population. Therefore, understanding how stigma affects PWIDs’ intrinsic and extrinsic motivation to change their substance use behavior and seek treatment is crucial. Furthermore, given the limited research on the intersection of various forms of stigma and the disparities in substance use among PWID, further research is warranted.

## Limitations and future research

Several limitations should be considered when interpreting these findings. First, the study’s cross-sectional design precludes causal inference. Thus, longitudinal studies are needed to delineate the temporal relationship between various factors that influence motivation to change substance use behavior among PWID. Furthermore, since primary data was collected between 2018 and 2021, perceptions of factors influencing PWIDs’ motivation to change substance use behavior may have evolved over the past 5 years. This underscores the need for research that examines the current context and time frame, which could be effectively addressed through a longitudinal study. Second, our reliance on self-reported data introduces the possibility of social desirability and recall bias. Third, the study sample was recruited from syringe service programs (SSPs) in a specific geographic region of Appalachian Kentucky. While this provided access to a hard-to-reach population, the findings may not be generalizable to PWID in other regions (e.g., urban areas) or to those not connected to harm reduction services. Fourth, using a single measure to assess motivation to change substance use behavior (i.e., how important it is for the PWID to cut down or stop substance use) may not capture the multifaceted nature of this construct. Future studies need to assess the interaction between the different types of motivation such as intrinsic and extrinsic motivation in influencing substance use behavior change. Nonetheless, evidence suggests that intrinsic motivation may be more potent in producing lasting change (41, 42). Fifth, categorization of experiences of violence as binary variable may oversimplify the severity of trauma. Therefore, future research should examine how different forms of violence, both collectively and cumulatively, influence motivation to change substance use behavior. Sixth, the study utilized the Substance Use Stigma Mechanisms Scale (SU-SMS), which emphasizes family and healthcare stigma. Future research on CPA should also explore other intersecting stigmas, such as community stigma. Seventh, while CPA was a significant predictor, it accounted for only a small portion of the variance in the MCSUB. This could be improved by using more robust statistical methods that consider confounding factors that may mediate or moderate the association between CPA and MCSUB. Eighth, our regression analysis did not control for the location of the syringe service programs (SSPs), as both were situated in a rural county serving PWID from similar socio-demographic backgrounds. Future studies should adjust for potential moderators, including the hours of operation of the SSPs, policy and practice guidelines, funding, and connections to peer or community services that can influence MCSUB. Finally, our measure of CPA focused solely on PWID who viewed their hometown as rural; hence, the findings do not include perspectives from those who considered their hometown urban. Additionally, CPA as a measure of belongingness may not capture meaningful connections for PWID who are transient, estranged from their hometown, or associate it with trauma. Therefore, future studies would benefit from employing a more comprehensive measure of belongingness that accounts for various conceptualizations and operationalizations of the term ‘connectedness’ or ‘belongingness’, which may include exploring both trait belongingness (where belonging is seen as a fundamental psychological need) and state belongingness (which relates to situation-specific feelings of belonging) (5).

Despite these limitations, this study has significant implications for prevention, policy, and clinical practice. From a prevention perspective, our findings suggest that community-level interventions that foster a sense of belonging or place identity could serve as protective factors for motivation to change substance use behavior. For policy and decision makers, these results highlight the need to implement integrated care models that address SUD comorbid factors such as MHD and trauma. For clinicians, assessing PWIDs’ existing place attachment or identity may be a critical component of the substance use treatment plan.

## Conclusion

This study highlights a significant association between community place attachment (CPA) and motivation to change substance use behavior among PWID. Enhancing CPA as a key social asset may enhance PWIDs’ perceived importance in cutting down or stopping substance use. Furthermore, addressing barriers related to co-occurring issues, such as mental health disorders and trauma from experiences of violence, is crucial in enhancing intrinsic motivation to change substance use behavior among PWID. The findings from this study emphasize the need to enhance CPA while simultaneously integrating care for substance use and mental health disorders, as well as incorporating trauma-informed care in tailored interventions and policies for PWID. This comprehensive approach is essential for motivating PWID to change their substance use behavior and to utilize substance use treatment services.

## Data Availability

The data analyzed in this study is subject to the following licenses/restrictions: for purposes of confidentiality the data sets will not be included. Results are only presented in aggregate form. Requests to access these datasets should be directed to hilary.surratt@uky.edu.
